# Data-Driven Insights into Porphyrin Geometry: Interpretable AI for Non-Planarity and Aromaticity Analyses

**DOI:** 10.1021/acs.jcim.5c00518

**Published:** 2025-04-20

**Authors:** Shachar Fite, Zeev Gross

**Affiliations:** Schulich Faculty of Chemistry, Technion—Israel Institute of Technology, Haifa 32000, Israel

## Abstract

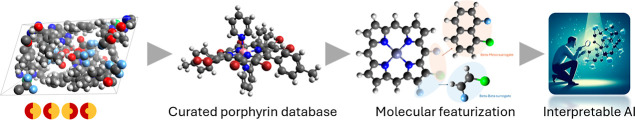

Porphyrins are involved in numerous and very different chemical and biological processes, due to the sensitivity of their application-relevant properties to subtle structural changes. Applying modern machine learning methodology is very appealing for discovering structure–activity relationships that can be used for design of tailor-made porphyrins for specific purposes. For achieving this goal, a high-quality set consisting of 425 metal porphyrins was established via curation of 7590 porphyrin structures from the Cambridge crystallographic database. Using data-driven techniques for analyzing nonplanarity and “structural aromaticity” allowed for validation of common knowledge in the field as well as discovery of new relations. Aromaticity was found to be influenced differently by distinct nonplanar distortions. Nonplanarity is more sensitive to macrocycle substitutions than to metal or axial ligand effects, while ruffled distortions are dominated by axial ligand size and metal properties. These findings offer new insights into structure–property relationships in porphyrins, providing a data-driven foundation for targeted synthesis to tune aromaticity and nonplanarity. Despite data set limitations, this work demonstrates the value of machine learning in uncovering complex chemical trends.

## Introduction

Tetrapyrroles, the “pigments of life”,^1^ are arguably the most researched and applied group of macrocyclic compounds to date.^2−4^ Among them, porphyrins stand out due to their crucial roles in diverse biological processes, such as oxygen transport and photosynthesis. N4-macrocyclic metal complexes are also widely applied for cancer treatment, molecular electronics, catalysis, and more. Despite of centuries long research activity, some of their fundamental properties are still not fully predictable.^5^ The design of new molecules geared toward specific applications is still very much dependent on trial and error.

The main structural feature of porphyrins is a large conjugated π-electron system, which is responsible not only for their colors but also for involvement in redox events occurring during catalysis.^5,6^ The Gouterman 4-orbital model^7^ was originally developed and eventually also well adapted for describing the electronic spectra of porphyrins. Extension of the model can be used to describe other photophysical properties, interactions with the chelated metal and its coordinated ligands, as well as redox properties of both the macrocycle and the metal. Aromaticity^8,9^ and nonplanarity^10,11^ are two terms often discussed in the context of conjugated π systems present in the porphyrin macrocycle.

Aromaticity is generally associated with thermodynamic, structural and magnetic properties, as well as electron structure and chemical reactivity.^12,13^ This is certainly relevant to porphyrins, whose aromaticity is more complex and very easily affected by various factors.^9,14−16^ Quantitative statements are particularly challenging for porphyrins as they have both local and global aromatic effects.^5,14,17^ The dominant Hueckel aromatic pathways are termed “bridged-annulene”^17^ for free-base and “internal cross”^18^ for metalated/deprotonated porphyrins. The former is an 18-π electron pathway involving 16 of the 20 C and 2 of the 4 N macrocycle’s atoms, while the latter contains only 12 C atoms and all 4 N atoms. Nevertheless, almost all computational works in the topic concluded that all atoms in the macrocycle participate in the aromaticity.^14^

Conjugated π-systems are often planar, which facilitates the delocalized electron distribution and confer unique electronic properties. Deviations from planarity are still frequently observed and are often considered as the source of significant changes in properties. Notably, most biologically relevant porphyrins are not planar.^2,11,19^ Nonplanarity in protein-conjugated porphyrins is believed to be one of the main ways to regulate activity. This might be exemplified by Hemoglobin and Cytochrome C, in which nonplanarity affects the binding selectivity to different gaseous molecules (oxygen vs CO vs CO_2_) and control of redox properties, respectively. This is also true for synthetic porphyrins, where nonplanarity induces red-shifted adsorption spectra and affects excited state properties.^11^

Despite this, there are few reports on the relationship between aromaticity and nonplanarity in porphyrins. Intuitively, nonplanarity would be expected to decrease aromaticity, as it weakens the π–π interactions. However, even for rather simple molecules, such as polyaromatic hydrocarbons (PAHs), the relationship is more complex. Dobrowolski et al.^20^ showed that the angle between rings in substituted naphthalene and anthracene is correlated with aromaticity metrics of each ring and global aromaticity. Krygowski^13^ studied similar naphthalene structures but found a less prominent decreasing relation. Shishkin^21^ uncovered a strong correlation between the lowest frequency of nonplanar deformation and few metrics of aromaticity, while demonstrating that the typical deformation energy of heteroaromatic benzene rings is quite low, suggesting small effect on aromaticity. This conclusion was supported by Radenković et al.^22^ showing that for PAHs, aromaticity is significantly more affected by the structure of the π system than by nonplanarity. The situation regarding porphyrins is more challenging, since quantitative description of aromaticity is more intricate therein. Determining relationships between aromaticity, nonplanarity, and structural variations is important not only for basic understanding, but also for designing porphyrins with desired properties.

Machine learning and statistical algorithms are increasingly used for gaining new insights into structure–activity relationships.^23−27^ The applications in the field are very wide and are reviewed thoroughly in ref (28). In porphyrins, there are few works using machine learning for property prediction and virtual screening,^29−32^ mainly based on calculated rather than experimental data.^33^ The models used in these works are often “black box” models, lacking option for human interpretability. Large, calculated databases are available for dye-sensitized solar cell applications,^34,35^ and much reported machine learning publications are for this specific application. Explainable models for porphyrins are particularly difficult as most interpretable representations in the field are based on feature vectors (descriptors), that often have only global features. From global features, it is quite difficult to isolate the effect of modification of the porphyrin macrocycle, which is the most common synthetic strategy for property tuning.

In this work, using a highly curated metal-porphyrin data set from the CCDC,^36^ the relationships between the structure, aromaticity, and nonplanarity are analyzed using interpretable machine learning technique. Metal porphyrins are the most relevant biological systems and for applications, and hence the focus was on them. Using a substituent-based representation, the relationships between substitutions and the properties were analyzed. This identified the most effective ways to influence nonplanarity and uncovered aromaticity to be affected by the different modes of nonplanarity in distinguished fashions.

## Methods

The CCDC^36^ is a large proprietary database containing millions of crystal structures from the published literature. This work describes the generation of a new database, which is based on carefully selected (vide infra) structures from the CCDC, combined with metrics of aromaticity and nonplanarity. It is important to emphasize that the calculated properties rely solely on the experimental geometries and do not contain any ab-initio results. The following steps were applied: (1) parsing CIF files into molecular format, (2) filtering out molecules by specific structural criteria, (3) augmenting the data with calculated properties, such as aromaticity and nonplanarity metrics, and (4) training and interpreting machine learning algorithms.

### Parsing and Filtering

7590 structures matched the query “porphyrin” of the CCDC. The crystal structures (CIF) from the database were converted to molecular format (XYZ) using pymatgen.^37^ In disordered structures, the more occupied position was taken, structures without a dominant occupancy were not used. That reduced the suitable structure count to 3206. The resulting molecules went through the following tests:

1 Has a central elemental ion (“metal”): that “metal” must be connected to all 4 pyrrole N atoms.
2 Connected molecule: the molecule does not have any nonconnected segments.
3 Monomer: the molecule has exactly one porphyrin macrocycle in it.
4 Proper saturation: each carbon or nitrogen atom in the macrocycle has exactly three connections, counting double bonds as a single connection.
5 Each substituent has only one connection to the macrocycle or the metal: multidentate substituents are excluded.
6 All components in the crystal structure have an even number of electrons.
7 Counter ions/molecules do not have metal atoms and have at most 20 atoms.

Molecules that passed are ensured to be uncharged monomers with simple substituent binding and a metal center. Only 425 molecules passed all the filters leading to a strongly curated, high-quality set of structures. This curation allows for analysis of the effect of substituents only, ruling out any effect from charge, N-substitution, polymerization, etc. Note that these filters eliminate most if not all open shell metal complexes like the d^9^ copper porphyrins. All tests were implemented using OpenBabel.^38^

This work is based solely on the molecular geometries of porphyrins extracted from their experimental crystal structures, even though the molecular structure in solution might be somewhat different than the crystalline one. Plenty of computational studies^39−41^ on porphyrins show a very high quantitative agreement between solid state and gas phase/solution geometries, encouraging analysis on solid state geometries as empirically accurate.

### Data Augmentation

The basic crystal structure information was augmented by calculating metrics derived from molecular geometry only. The features that were calculated are divided into three categories: (1) aromaticity, (2) nonplanarity, and (3) substituent and structure descriptors.

### Aromaticity

Aromaticity can be defined in many ways, leading to many numerical descriptors for it. In this work only structural aromaticity metrics were used,^13^ as they do not require any quantum calculation. The most common structural descriptor is the harmonic oscillator model of aromaticity (HOMA) score. It assumes that all bonds in aromatic structures have approximately the same length. Accordingly, HOMA is defined as the mean square deviation from this ideal bond length. The ideal length of the bond depends on the identity of the bounded atoms. This definition allows for the HOMA score to be calculated on a whole aromatic system or any subsystems in it, thus quantifying both global and local aromaticity of complex systems using the same metric.
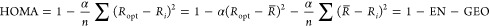
1

Equation 1 shows the definitions of HOMA, EN, and GEO. α is a normalization factor ensuring that the score will be between 0 to 1, *n* is the number of bonds in the system, *R*_opt_ is the ideal bond length in the structure, *R̅* is the average bond length, and *R*_*i*_ is the bond length. α was determined for each system to ensure that a planar, unsubstituted porphyrin will have score 1. The C–N bond treatment was done as detailed in ref (13). *R*_opt_ values for CC bonds were taken from there as well. EN and GEO are a decomposition of the HOMA score, giving more information about the factors causing lower score. EN is the factor stemming from bonds being on average shorter or longer than the optimal aromatic length. GEO shows for deviation from a constant bond length value. Despite its simplicity, HOMA was shown to have good agreement with more sophisticated metrics of aromaticity both in porphyrins,^17,18^ and in other compounds.^42−44^

**Figure 1:**
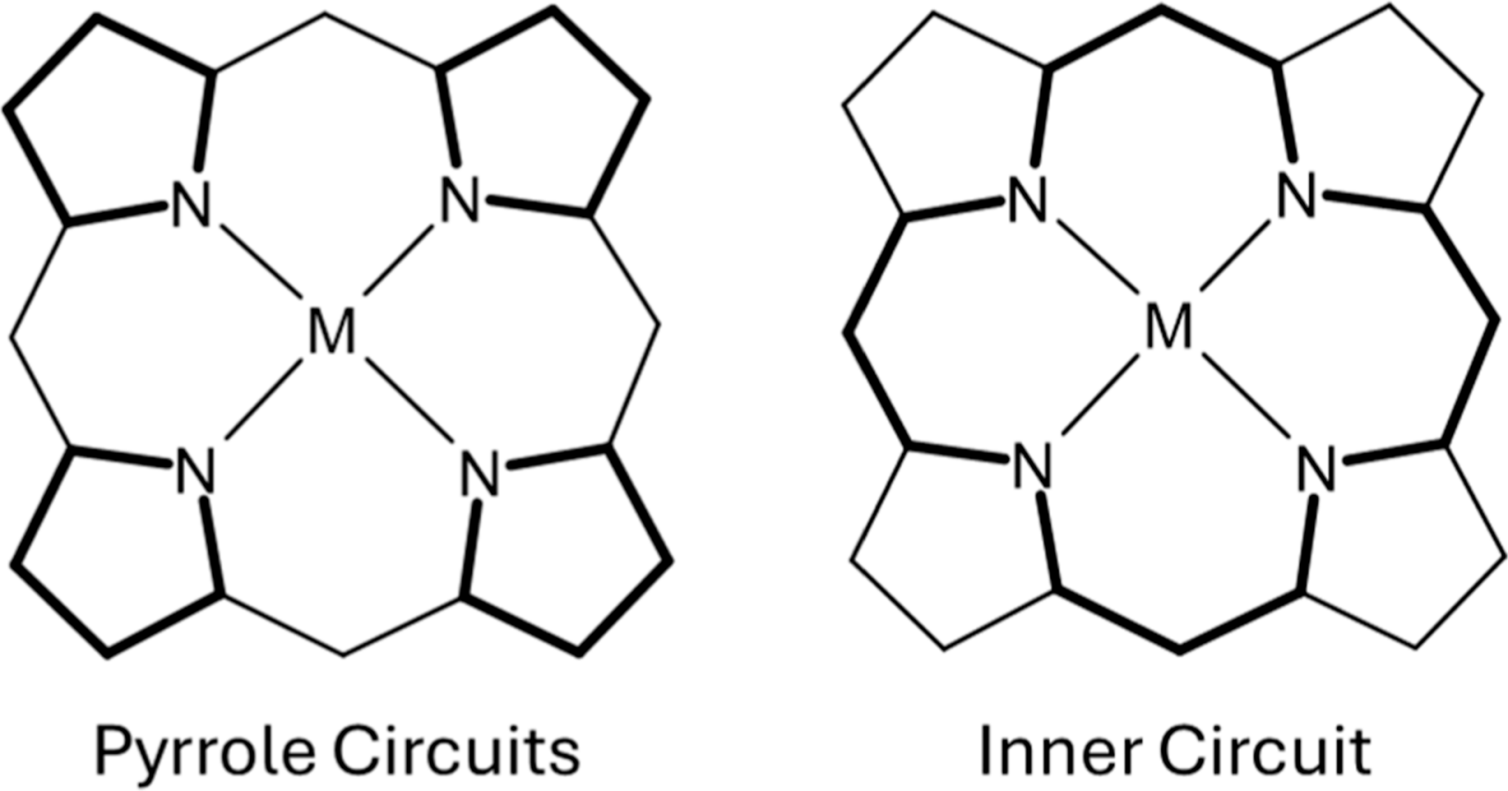
Circuits used for calculating HOMA scores on each structure.

As described in the introduction, there are specific pathways common for calculation in porphyrins. In metal porphyrins,^17,18^ it was shown that the “inner cycle” is best capturing the aromaticity of the system is correlated with the other aromaticity metrics. Additionally, we calculated the score for each pyrrole in the system, accounting for local aromaticity. The different cycles are presented in Figure 1.

Being a purely geometric metric, the HOMA score can be inaccurate for some strained geometries, as shown by Feixas et al. in their studies of bond-length alteration (BLA) deformations in benzene.^42,43^ However, the distortions in porphyrins are out-of-plane and therefore not BLA.^2^ Additionally, in this work, they do not have a very large size. This suggests the HOMA score as a very cheap and relatively accurate metric for estimating porphyrin’s aromaticity.

### Nonplanarity

**Figure 2:**
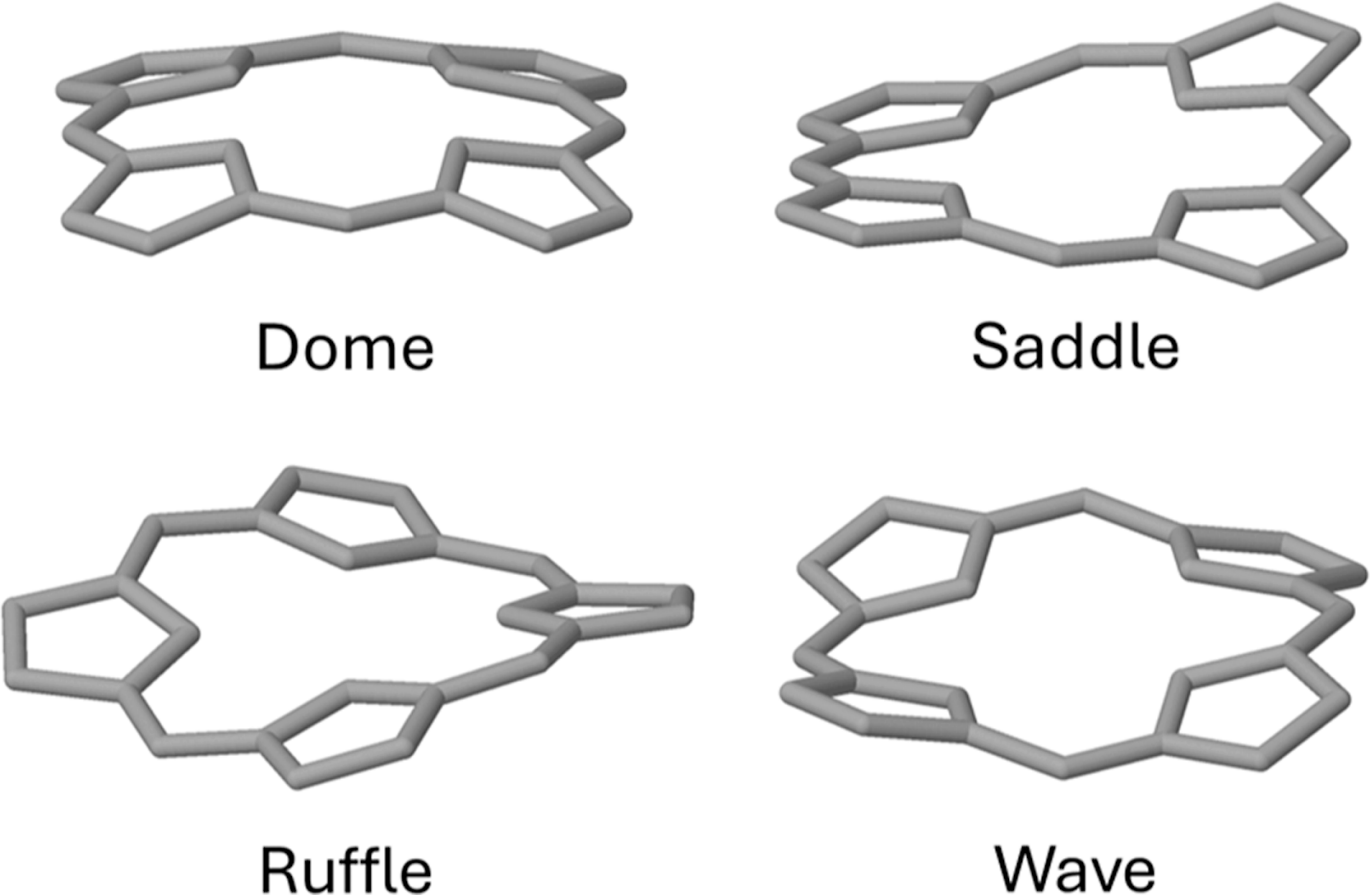
Principal nonplanar distortions in the porphyrin macrocycle.

The nonplanarity of porphyrins is usually described by natural structure decomposition (NSD) analysis.^2,11,45^ This analysis allows for decomposition of the total nonplanarity (deviations from the N4 plane) to different symmetries, such as ruffling, saddling, and so forth. These different symmetries determine the exact nature of the nonplanarity distortions, allowing us to better understand their effect. The principal distortions for the porphyrin macrocycle are depicted in Figure 2. NSD calculations were performed using PorphyStruct on all structures.^46^ The total out-of-plane deviation was taken, as well as the decomposition to the different modes. The modes can take a negative value depending on the geometry. This allows one to distinguish between mirrored distortions of the same mode. Throughout all the paper, unless stated otherwise, the absolute value of a mode is used.

### Substituent Descriptors

For the application of machine learning models on the database, a descriptor-based representation was used. The aim of the study is to learn the relationships between the substituent features to the aromaticity and nonplanarity of the macrocycle. The feature vector of the molecule must contain information only on the substituents. There are plenty of techniques for substituent or ligand features that are geometry based.^47^ The most common ones are based on the so-called “cone angles” of a ligand or substituent with respect to the bounded atom. Generally, cone angles are a measure of ligand’s size by calculating the angle of a cone containing it. The larger the cone angle, the bulkier the ligand. The cone usually starts from the binding site of the ligand. In this work, the maximal coning angle was used as a descriptor for all macrocycle substituents and the axial ligands. The maximal coning angle is defined as the minimal angle of a cone that contains all the substituents, originating from the bounded atom. Note that this representation is useful only when the substituents are monodentate, signifying the importance of the strict filtration used in this work.

The feature vector of each structure is a vector containing the coning angles for all ring substituents, the central metal radius, and the coning angles of the axial ligands if any exist. Substructure matching for the macrocycle ring ensured that the representations for all molecules are symmetrically equivalent—reducing bias due to the ordering of the vector.

**Figure 3:**
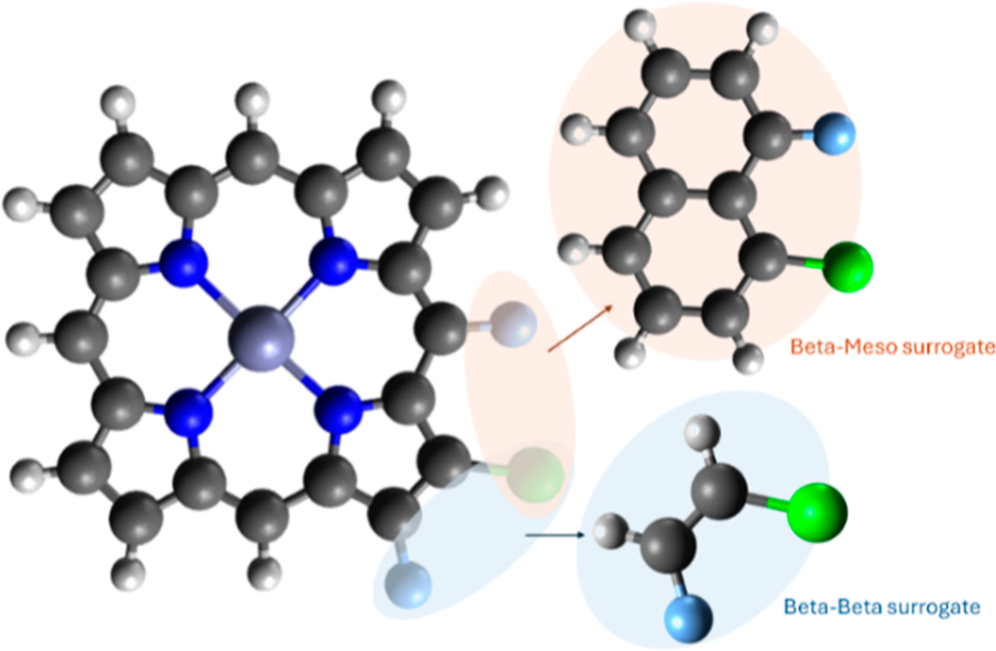
Surrogate systems used for distance measurements.

The crowding of different substituents on the porphyrin macrocycle is a very common synthetic approach for generating nonplanar porphyrins.^11^ The coning angles do not express crowding inherently; only correlation between them might suggest a high crowding. To overcome this, the minimal distance between neighboring substituents was calculated for each pair in the data set. To avoid inserting information about the macrocycle in the representation, these distances were calculated on a surrogate system (Figure 3). This distance metric allows for the model to learn directly from the crowding, thus providing each crowding mode an influence score.

### Machine Learning Details

The models were trained using scikit-learn.^48^ The data were split into 395 training structures (90%) and 30 testing structures (10%). Considering the relatively small data set size, and thus limited generalizability of the models, special care was taken in testing and training. Train and test structures are selected to ensure that test set structures contain at least one substituent on either beta, meso, or axial position, not present in the training set. This ensures that the trained models can predict properties on previously unseen substituents. Performance was estimated on the test set. Two models were used in this work; the first is the LASSO model, chosen for its great simplicity and interpretability. The second is the nonlinear random forest (RF) model (RF). The former is simpler and has higher bias, while the latter has less bias and can uncover more complex trends in the data, with an increased risk of overfitting. The regularization parameter for the LASSO model was selected via five-fold cross validation. Each molecule was represented using a descriptor vector containing metal radius, metal coordination number, and mean axial ligand cone angle. Additionally, the “cone angle” representation used the mean cone angle values of substituents in meso and beta positions. The “distances” metric used mean beta–beta substituent distance and mean meso–beta substituent distance. The descriptor details are given in the Substituent Descriptors section above. In total, there are five descriptors for each molecule in each representation. All the metrics presented are mean values of 10 bootstrap experiments with a confidence interval of 95% unless stated otherwise.

## Results and Discussion

### Data Exploration

**Figure 4:**
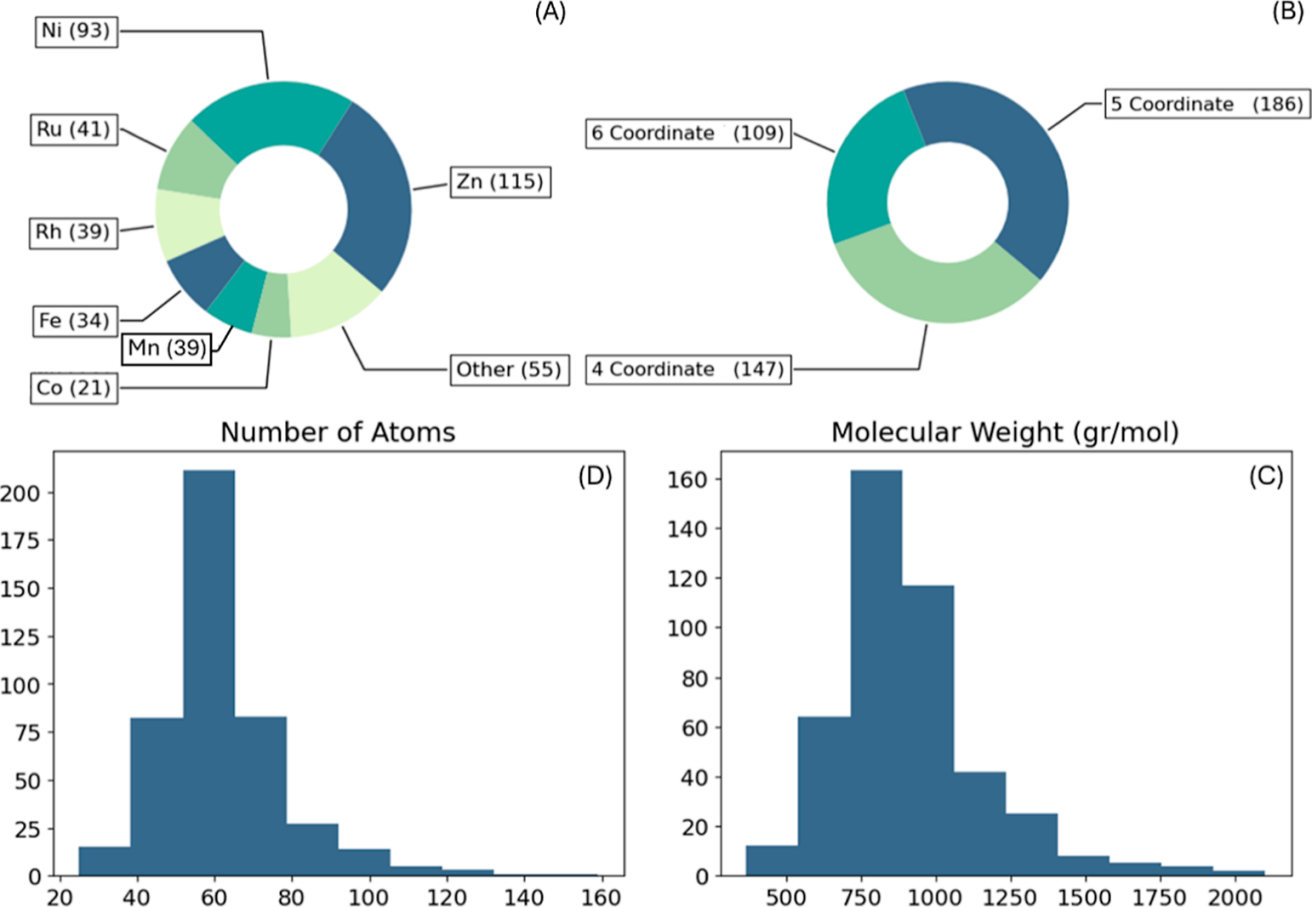
Description of the database. Distribution of (A) metal centers, (B) coordination numbers, (C) number of atoms, and (D) molecular weight.

The database consists of 425 metal-porphyrin structures. There are 23 different central metals in the database, but the majority (67%) are structures of zinc, nickel, ruthenium, or rhenium porphyrins (Figure 4A). The coordination number of the structures is most commonly 5 (44%), followed by 4 (35%) and 6 (26%), demonstrating a relatively even distribution (Figure 4B). Looking at the structure sizes, most structures have up to 100 atoms and 1000 g/mol molar mass showing that they do not have any particularly large substituents, proving the curing process successful (Figure 4C and D).

The degree of substitution in the structures is presented in Table S1, revealing that the most common pattern is no beta substitutions (H) and four meso substitutions (62%) and, after this pattern, eight beta substitutions with no meso substitutions (8%) and full macrocycle substitution (8%). This is quite representative of the research activity in porphyrins, which largely focuses on meso tetrasubstituted derivatives.^5,49^

**Figure 5:**
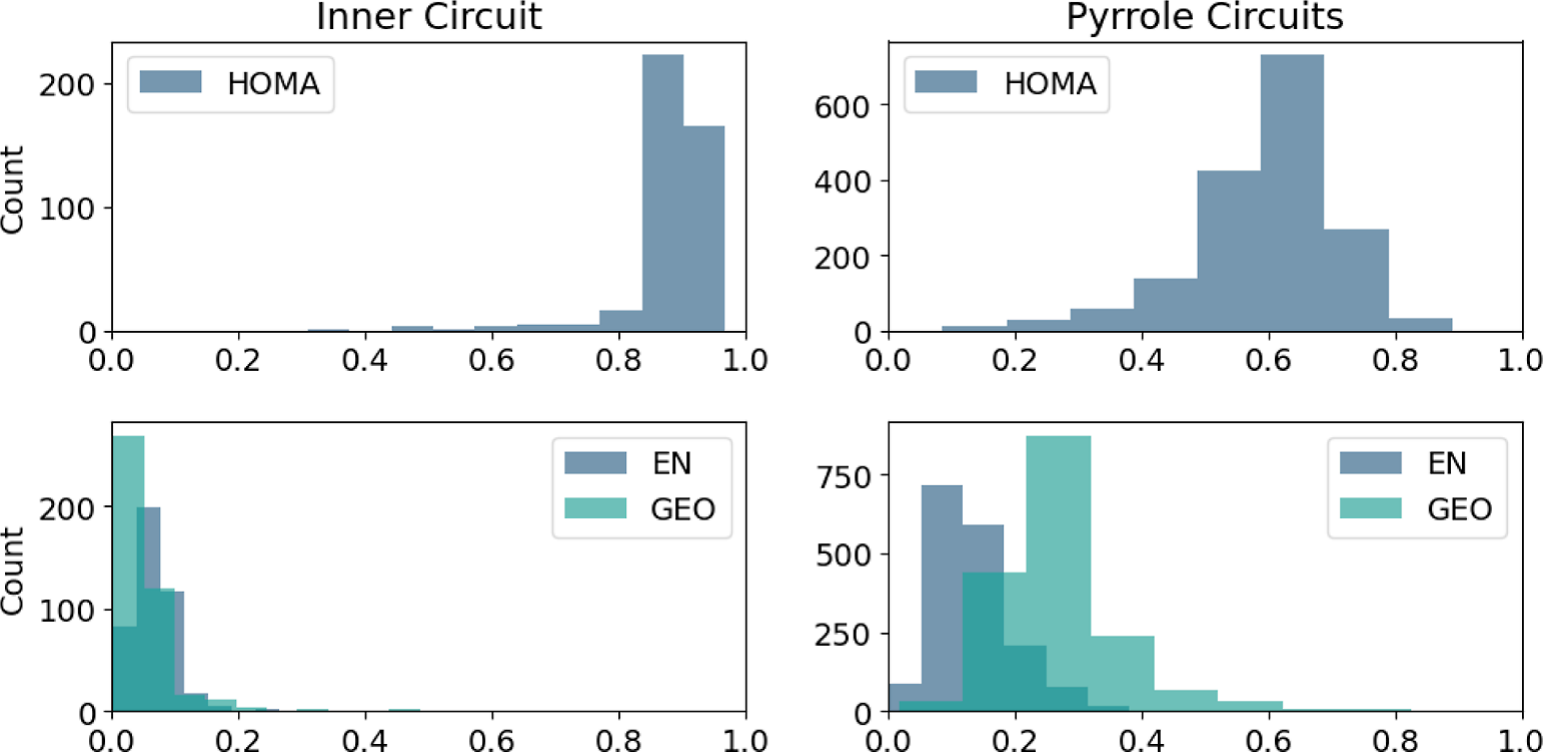
Distributions of the HOMA score, EN, and GEO for inner circuit and pyrrole circuits.

Both local and global aromaticity were analyzed for each structure. The global aromaticity was estimated using the “inner cycle” of the macrocycle and the local aromaticity was estimated by the pyrrole ring (see Methods section). As seen by ref (18), the HOMA for the inner cycle is greater than the HOMA for the pyrrole rings (Figure 5, top plots), suggesting that aromaticity is better described by global and not local effects. Note that this result is in seeming contradiction to results on free-base porphyrins where the pyrroles seem to have more pronounced aromaticity compared to the macrocycle.^14^ This aspect has already been addressed by Schleyer et al.^17^ by pointing out that free-base and metalated porphyrins differ in terms of relative contributions of the pyrroles’ Kekule structures leading to less aromatic pyrroles in the latter case.

Judging by the EN and GEO values of the inner circuit (Figure 5, bottom plots), the former is dominant and affects aromaticity more than the latter. For the pyrrole rings, the values are significantly more different, with GEO being the dominant factor.

**Figure 6:**
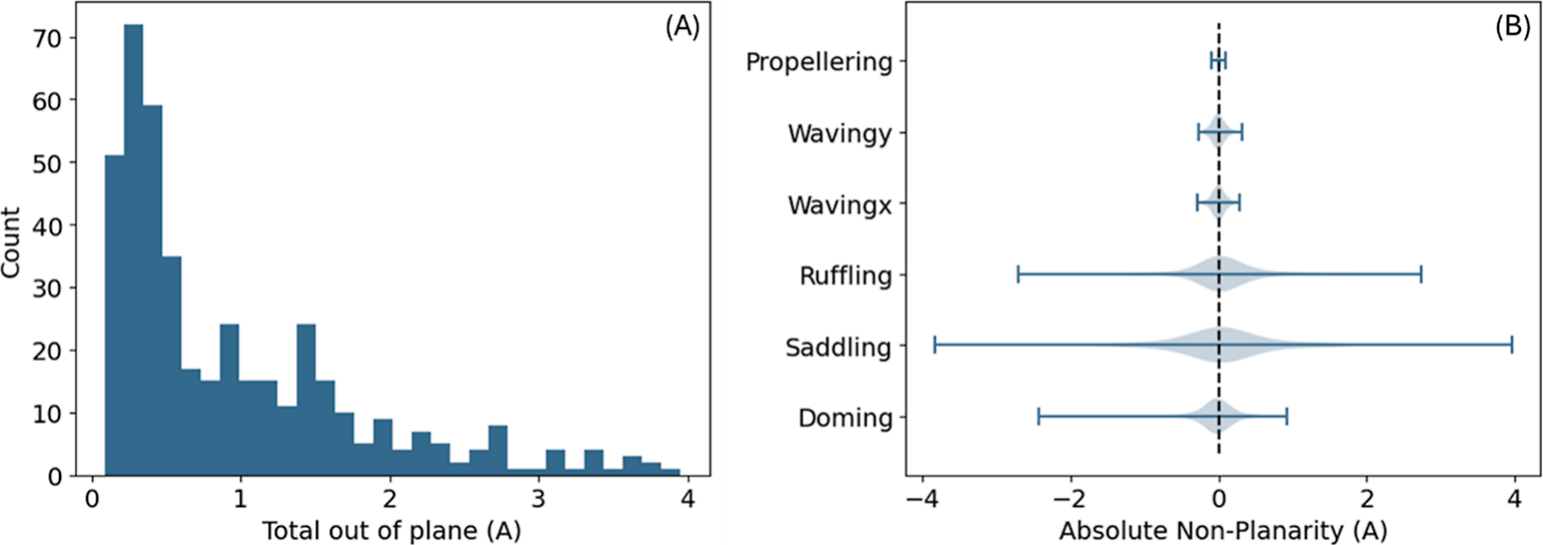
Distribution of total out of plane in angstroms (A) and of absolute nonplanarity in each mode (B).

The total out of plane metric of each structure has a median of 0.6, or 0.025 Å mean deviation per atom (Figure 6A), small enough for considering about 50% of the structures as quite planar. Looking at the different modes of nonplanarity (Figure 6B) reveals that only ruffling, saddling and doming modes have high values. These are apparently the three dominant nonplanarity modes in porphyrins, consistent with common knowledge.^2,11^

### Relation between Aromaticity and Nonplanarity

One of the basic criteria used in teaching aromaticity is the planarity of the particular macrocyclic system, which is required for best overlap between the involved p orbitals. Intuitively, the more planar the structure, the more aromatic it is. This rule of thumb does not necessarily hold for conjugated aromatic systems such as PAHs, as outlined in the introduction. More complex relationships might be expected for porphyrins because of the intricate structure of the macrocycle that also contains heteroatoms (nitrogen) and metal ions. This work was started by searching for correlation between the HOMA scores and the total out of plain and both the inner circuit and pyrrole HOMA scores.

**Figure 7:**
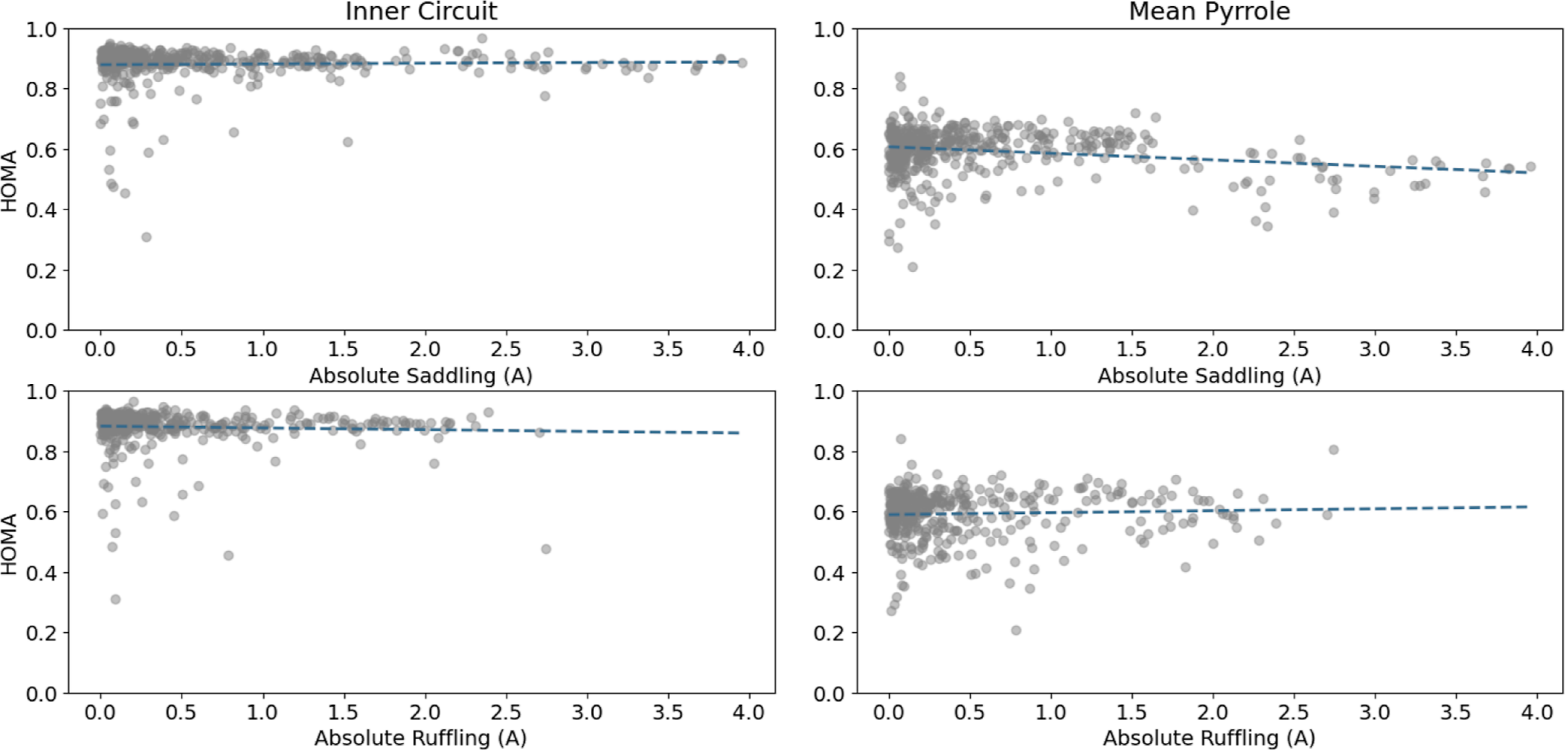
HOMA scores in different circuits vs the absolute non planarity of saddling and ruffling. The lines are best-fit lines for each set meant to guide the eye.

The total nonplanarity has been decomposed into all the above-discussed modes. The two modes, ruffling and saddling, that are most frequent were examined for correlations. Results depicted in Figure 7 disclose that saddling or ruffling distortions influence the aromaticity score differently. The inner cycle HOMA score, representing global aromaticity, is affected similarly by the different modes and has a slightly decreasing relation. This is not the case for the average pyrrole ring HOMA score, representing the local aromaticity: saddling distortions lead to a sharp decrease in local aromaticity, while ruffling distortions seem to increase the local aromaticity. Finding a nonplanar distortion that increases the aromaticity of the structure is quite a surprising phenomenon.

Further corroboration has hence been examined by starting with a planar porphyrin macrocycle and imposing pure distortions (e.g., only ruffling or saddling) while looking at the HOMA scores (Figure S1). In three out of the four cases, the expected inverse relationship appeared, while there was a region with positive relationship between the ruffling mode and the pyrrole HOMA score, up until a total out of plane deviation of about 2 Å. This is in nice correlation with the conclusions drawn from the experimental data.

The EN and GEO scores are plotted versus the absolute nonplanarity of each mode in Figure S2. The figure shows that the EN increases with increasing distortion, while the GEO is almost not affected. This suggests that bond elongation is responsible for the decrease in aromaticity, in all tested nonplanarity modes and circuits.

### Machine Learning

**Figure 8:**
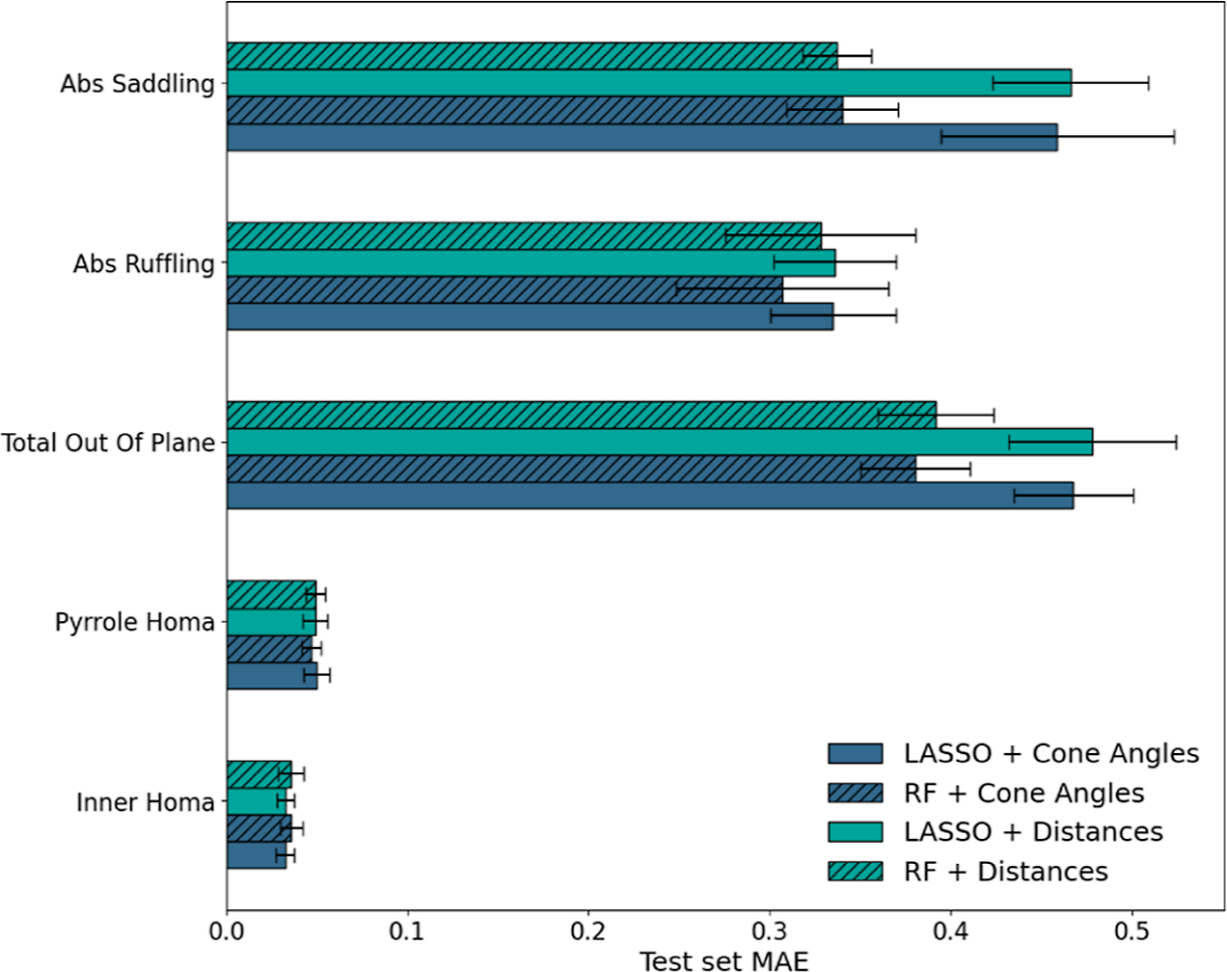
Mean absolute error values on the test set for LASSO and RF models with both cone angles representation and distance representation. Error bars represent confidence intervals with *p* = 0.95 from 10 bootstrap experiments.

The linear model with regularization (LASSO) and RF model were trained as described in the Methods section. The test set errors of the models with the two representations are shown in Figure 8, uncovering that both models with both representations have similar performance on all target properties. Detailed model fitting parameters and additional performance metrics are provided in the Supporting Information (Tables S2–S5).

For nonplanarity metrics—total out of plane, saddling, and ruffling—the absolute error is approximately 0.45 Å. This is equivalent to average atomic dislocation of about 0.02 Å, therefore very close to the experimental error in crystal structure solving. Doming was not included in the predicted properties as the number of domed molecules in the database is quite small (Figure 6B), thus prohibiting true prediction. The performance of the LASSO and RF models is quite similar, encouraging the use of the simpler and more informative LASSO model for further use (see below).

In the case of HOMA, the models achieved moderate errors around 0.05, with relative errors below 10%. This level of accuracy is barely sufficient for the purposes of model interpretation. The comparable performance of both the coning angle and distance-based descriptors suggests that either metric can be effectively employed for predicting structure–activity relationships in porphyrins. This interchangeability highlights the flexibility of the model in utilizing different structural descriptors without compromising predictive accuracy. Overall, the results suggest that the proposed feature sets with the LASSO model provides a satisfying model to be used for further interpretation.

**Figure 9:**
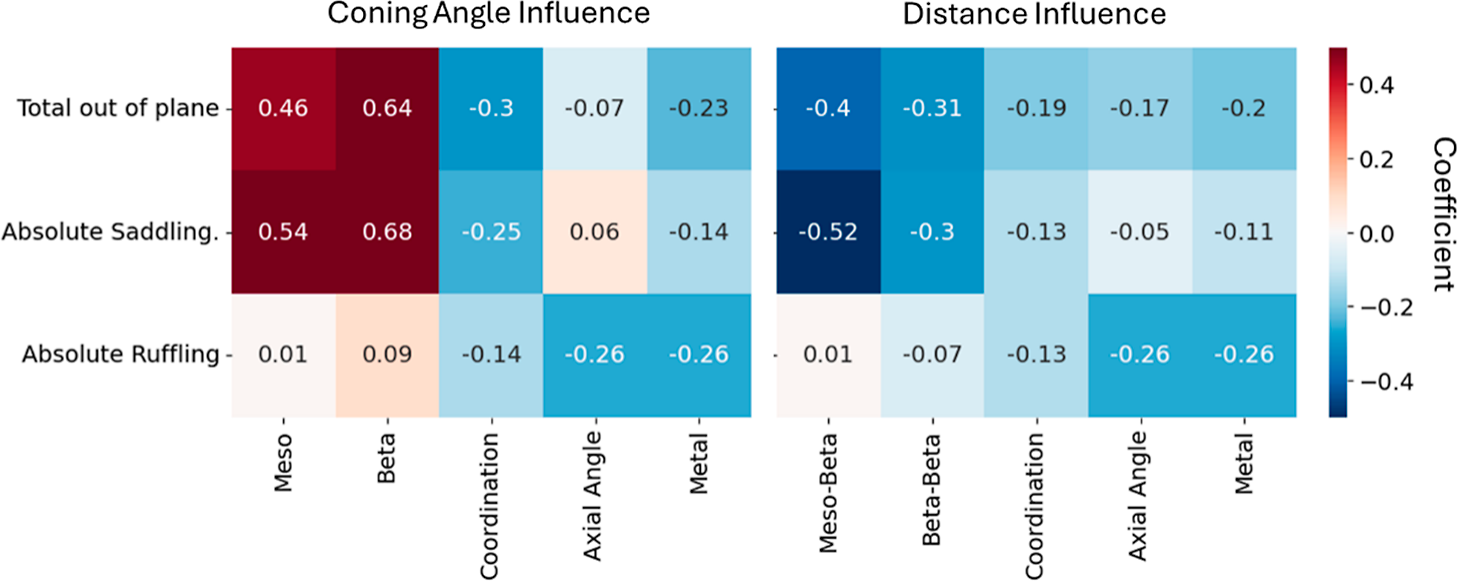
Feature importance scores for both (A) coning angle descriptors and (B) distance descriptors.

The LASSO models used in this study provide a linear relationship between the structural features and the target properties. Using the coefficients of the different features, their influence can be estimated. Figure 9 presents a heatmap of the coefficient values for each representation. Note that models predicting HOMA scores are not interpreted due to their relatively high error. Full coefficient information, including confidence intervals, is available in the Supporting Information (Tables S6 and S7). A RF model can also be interpreted to get feature importance, such an analysis was performed, and very similar results were achieved. Full details are given in the Supporting Information.

Examining the coefficients of the representations reveals that the various modes of nonplanarity are governed by different substitutions. Saddling is affected mostly by macrocycle substitutions and ruffling is more sensitive to axial ligand angles and metal size. This result aligns with existing literature about crowded macrocycles in which saddling is the dominant mode.^11^ In general, it encourages the idea of possible design principles for preparing porphyrins with targeted nonplanarity. The total nonplanarity is affected by both the axial ligand, macrocycle substituents and metal, with general similar behavior to the saddling mode.

A closer look at the results shows that for all nonplanarity modes, bulkier substituents, having larger cone angles do lead to less planar structures (positive coefficients). More crowded macrocycles, having lower intersubstituent distances, will also be less planar (negative coefficients). For saddling modes, the coefficients of beta and meta coning angles are similar, alluding that their effect is similar. The meso–beta distance has a larger absolute value than the beta–beta distance, suggesting that crowding on these positions has more effect on saddling.

The coefficients of metal radius are negative, meaning that larger metal centers lead to more planar structures. This somewhat unintuitive result is supported by literature on porphyrins with large metal centers.^11,50^ The “planarizing” effect of large metals stems from their binding to the 4N core limiting the distortion range with larger metal centers. Metal size has larger coefficients for ruffling compared to saddling, indicative of a larger planarizing effect in the former.

The coefficients of the coordination sphere and the mean axial cone angle reveal again an inverse relationship. Larger axial ligands lead to more planar structures. Bigger coordination spheres also lead to more planar structures. There is a correlation between the two metrics and therefore their effect is inseparable. The correlation stems from the fact that when the coordination number is 4 (no axial ligands) their angle is fixed to −1 in the representation. Another correlation exists between the metal radius and coordination number, as larger metals tend to require larger coordination sphere. From a steric perspective, a very large axial ligand, symmetrically bound to the metal (coordination number = 6) might prohibit large nonplanar distortions in the macrocycle.

Note that the effect on nonplanarity might not necessarily be linear for intermediate values of axial ligand cone angles and coordination number. Ruffling, which is a less common, but still very important property-affecting mode, is predicted by the model to be induced via macrocycle substitution but almost nullified by large metals and coordination numbers (Figure 9, bottom row). This might explain why the prototypical ruffled complexes are nickel porphyrins, i.e., very small metal ion and small affinity for axial ligands, due to the low-spin d^8^ configuration.

## Conclusions

In this study, a comprehensive analysis of the geometric properties of porphyrins was conducted using the CCDC database, resulting in the curation of 425 metal porphyrins to create a high-quality set of crystal structures for analysis. The primary focus was on examining the nonplanarity and structural aromaticity, as expressed by HOMA, of these porphyrins, and investigating their relationships with substituent structures as well as with each other.

Analysis of the data set revealed a bias toward lighter d-block metals, such as zinc and nickel, and showed that more than half of the structures could be classified as planar. Among the nonplanar structures, the majority exhibited saddling and ruffling distortions, with only a few showing domed configurations. When comparing HOMA scores with total nonplanarity, no clear trend emerged, though a slightly decreasing relationship was observed. However, a detailed examination of HOMA versus specific nonplanarity modes revealed that the local aromaticity of porphyrins (pyrrole HOMA) is influenced differently by various modes, decreasing with saddling and increasing with ruffling.

Using a LASSO model combined with a substituent-based molecular representation, this study uncovered the relationship between substituents’ steric effects and the geometric properties of porphyrins. Total out-of-plane and saddle distortions were found to be heavily dependent on macrocycle substitutions, with meso–beta playing a more significant role than beta–beta crowding. The ruffled distortions, however, were predominantly influenced by coordination number and size of both the metal and its axial ligand(s), in line with established findings. Coefficients of the different steric descriptors were analyzed to provide further insights on the effect of each individual transformation. This helped in revealing both well established and novel trends.

This study not only provides empirical support for “common wisdom” in the field but also introduces new insights into the structure–property relationships in porphyrins. The findings have practical implications, suggesting that porphyrin synthesis could be directed toward achieving targeted nonplanarity or aromaticity values by changing substituent size, crowding, metal size or coordination number, thereby aiding in the precise tuning of these properties. Additionally, this research highlights the potential for extending the analysis to electronic or other nongeometric properties, exploring nonsteric substituent effects, and examining free base porphyrins.

Despite the significant insights gained, several limitations of the study should be acknowledged. The relatively small database led to some biases in the results, particularly the prevalence of planar structures and limited representation of distortion modes. There was also a bias toward popular metal centers, particularly lighter d-block elements. The limited data set necessitated the use of simpler machine learning methods to avoid overfitting; however, access to more data could enable the application of more sophisticated modeling techniques.

Finally, this research represents a notable contribution to the field of data-driven discovery, as it is, to the best of our knowledge, the first study to provide data-driven explanations for the trends in aromaticity and nonplanarity in porphyrins. It is certainly among the few studies that address substituent effects using a data-driven approach, thereby paving the way for future research that leverages large data sets and machine learning to uncover new insights in molecular chemistry. For example, it would be interesting to examine the influence of substituents on properties such as strength of diamagnetic current effect, as well as dictating reactivity/selectivity of aromatic substitution on the macrocycle.

## Data Availability

The code developed and used in this publication is available at https://github.com/ajr15/PorphyrinCrystalStructures. A list of the CCDC codes for all database structures is available in the Supporting Information.

## Supporting Information Available

Additional database statistics, HOMA for ideal distortions, full fit and feature importance details, and CCDC codes used to build the database (PDF)
