# Supplementary material for: Data-Driven Insights into Porphyrin Geometry: Interpretable AI for Non-Planarity and Aromaticity Analyses
Source: J Chem Inf Model. 2025 Apr 20;65(9):4403–11. doi: 10.1021/acs.jcim.5c00518 (PMC12076509; doi:10.1021/acs.jcim.5c00518)
Supplement: Supplementary file 1 — ci5c00518_si_001.pdf [file ci5c00518_si_001.pdf]

Supporting Information for
Data-Driven Insights into Porphyrin Geometry: Interpretable AI for Non-Planarity and Aromaticity Analyses

Shachar Fite and Zeev Gross

Schulich Faculty of Chemistry, Technion—Israel Institute of Technology, Haifa 32000, Israel

Substitution Patter Frequency

To check for the variance between structures in the database, the frequency of each substitution pattern was calculated. “Substitution” means no hydrogen so 0 beta substitutions mean all hydrogens in beta positions and 8 substitutions means no hydrogens in beta positions. The results are presented in Table S1.

Table S1: Frequency of different substitution patterns in the database

# Beta Substitutions	# Meso Substitutions	# Structures	% Structures
0	0	4	0.94
0	1	2	0.47
0	2	8	1.88
0	3	23	5.41
0	4	265	62.35
1	3	1	0.24
1	4	5	1.18
2	3	2	0.47
2	4	6	1.41
3	4	3	0.71
4	1	1	0.24
4	4	10	2.35
5	1	1	0.24
5	4	1	0.24
6	4	3	0.71
7	4	1	0.24
8	0	35	8.24
8	1	13	3.06
8	2	6	1.41
8	3	2	0.47
8	4	33	7.76

HOMA Relationship with Non-Planarity

Ideal Structure displacements

Calculation of HOMA scores was made on “ideally displaced” structures. To make such calculation, displaced porphyrins structures were taken from [1]. To generate structures with different displacements, their coordinates were centered, and their displacements from planarity where calculated. These displacements were scaled by some factor, to make the molecule less or more displaced. To make sure that the bond lengths are roughly preserved, the resulting atomic coordinates were normalized to preserve the average bond length in the molecule. The results for the displaced structure’s HOMA scores are in Figure S1. Similarly to real structures, the ruffled geometry “hinders” the deterioration of aromaticity for pyrrole rings compared to the saddled. While the effect on the inner circuit is similar for both distortion modes.

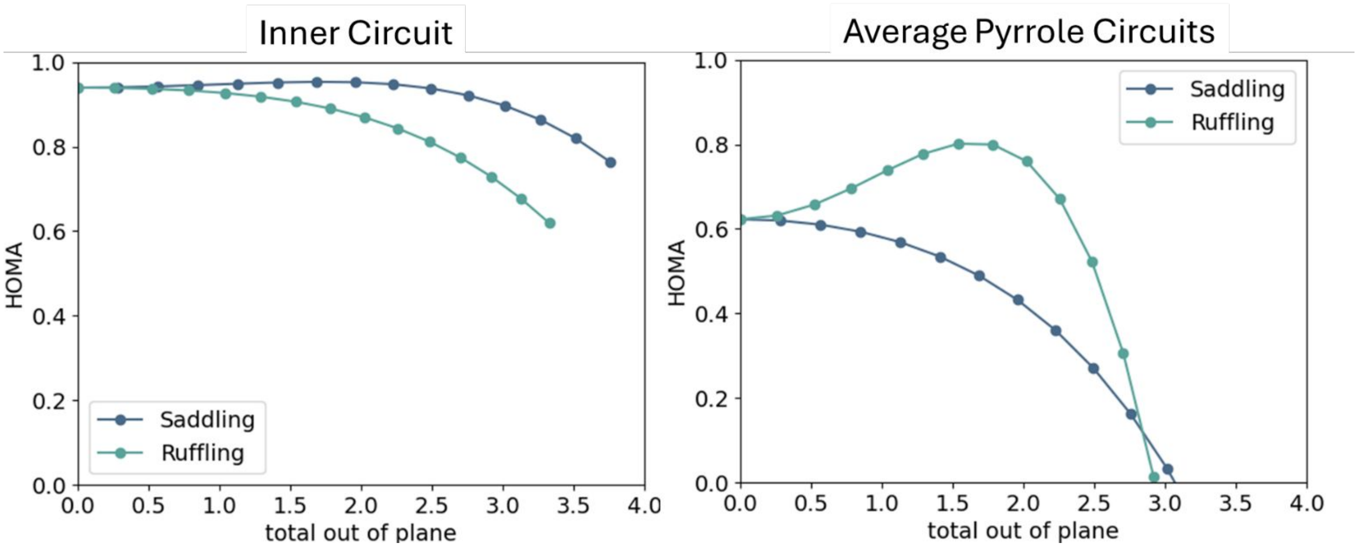

Figure S1: HOMA scores on ideally ruffled and saddled structures

HOMA Components vs. Non-Planarity Modes

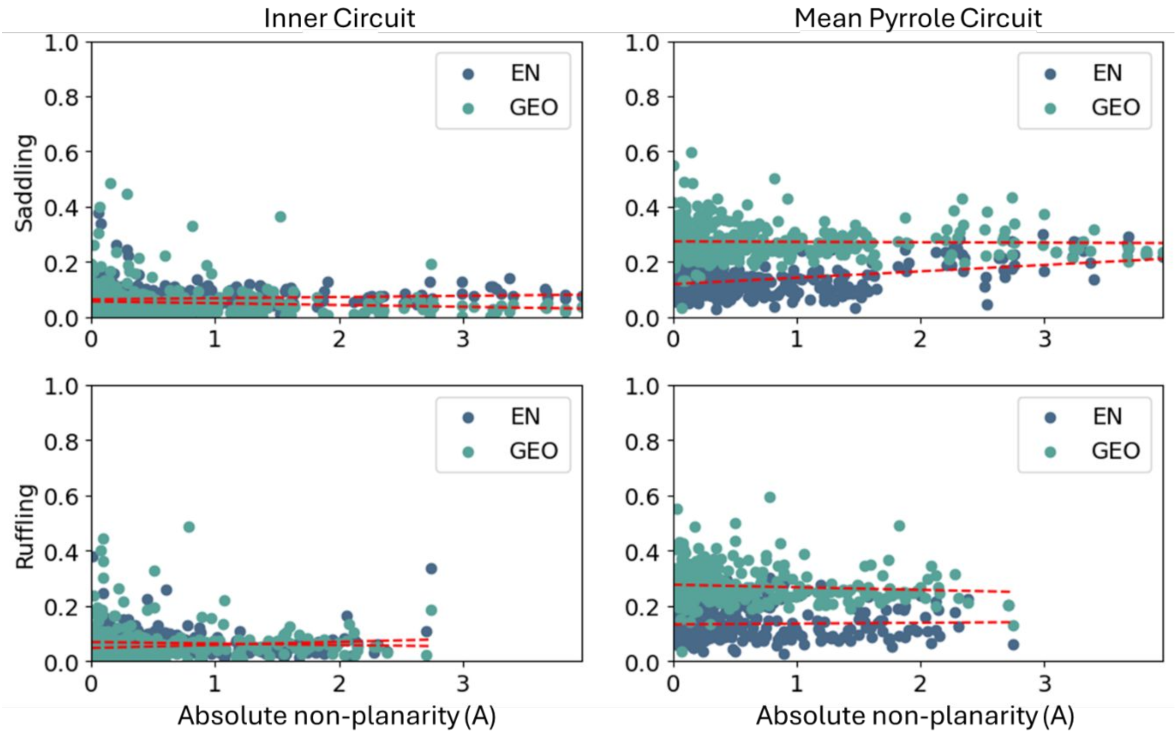

Figure S2: HOMA score for different circuits vs. the absolute non-planarity in saddling and ruffling

Full Model Details

Performance Metrics

Table S2: Full performance metrics for the LASSO model, from 10 bootstrap experiments, for the coning angle representation along with confidence intervals (α=0.05)

Property	MAE		RMSE		Pearson R^2	
	Train	Test	Train	Test	Train	Test
Absolute Ruffling	0.345±0.003	0.335±0.035	0.473±0.004	0.443±0.047	0.303±0.007	0.228±0.094
Absolute Saddling	0.51±0.005	0.459±0.064	0.659±0.006	0.606±0.083	0.365±0.009	0.369±0.131
Inner Circuit HOMA	0.034±0.001	0.033±0.005	0.067±0.001	0.054±0.015	0.013±0.004	0.041±0.034
Mean Pyrrole HOMA	0.046±0.001	0.05±0.007	0.068±0.001	0.071±0.015	0.207±0.01	0.256±0.123
Total out of Plane	0.488±0.002	0.468±0.033	0.62±0.002	0.602±0.033	0.459±0.007	0.391±0.114

Table S3: Full performance metrics for the LASSO model, from 10 bootstrap experiments, for the distance representation along with confidence intervals (α=0.05)

Property	MAE	RMSE	Pearson R^2

	Train	Test	Train	Test	Train	Test
Absolute Doming	0.116±0.002	0.328±0.052	0.172±0.002	0.468±0.077	0.926±0.002	0.236±0.119
Absolute Ruffling	0.129±0.001	0.337±0.019	0.182±0.002	0.443±0.037	0.958±0.001	0.519±0.19
Absolute Saddling	0.014±0	0.036±0.007	0.026±0.001	0.062±0.018	0.952±0.003	0.042±0.07
Inner Circuit HOMA	0.017±0	0.049±0.005	0.025±0.00	0.072±0.008	0.932±0.001	0.178±0.087
Mean Pyrrole HOMA	0.139±0.002	0.392±0.032	0.19±0.002	0.513±0.051	0.956±0.001	0.475±0.151
Total out of Plane	0.116±0.002	0.328±0.052	0.172±0.002	0.468±0.077	0.926±0.002	0.236±0.119

Table S4: Full performance metrics for the RF model, from 10 bootstrap experiments, for the coning angle representation along with confidence intervals (α=0.05)

Property	MAE		RMSE		Pearson R^2	
	Train	Test	Train	Test	Train	Test
Absolute Ruffling	0.115±0.002	0.307±0.059	0.175±0.003	0.437±0.088	0.925±0.002	0.291±0.117
Absolute Saddling	0.131±0.002	0.34±0.031	0.188±0.002	0.447±0.038	0.955±0.001	0.521±0.178
Inner Circuit HOMA	0.013±0	0.036±0.006	0.026±0.001	0.062±0.017	0.924±0.003	0.074±0.133
Mean Pyrrole HOMA	0.017±0	0.047±0.005	0.025±0	0.069±0.01	0.936±0.001	0.227±0.132
Total out of Plane	0.137±0.001	0.38±0.03	0.185±0.002	0.488±0.049	0.959±0.001	0.508±0.133

Table S5: Full performance metrics for the RF model, from 10 bootstrap experiments, for the distance representation along with confidence intervals (α=0.05)

Property	MAE		RMSE		Pearson R^2	
	Train	Test	Train	Test	Train	Test
Absolute Doming	0.116±0.002	0.328±0.052	0.172±0.002	0.468±0.077	0.926±0.002	0.236±0.119
Absolute Ruffling	0.129±0.001	0.337±0.019	0.182±0.002	0.443±0.037	0.958±0.001	0.519±0.19
Absolute Saddling	0.014±0	0.036±0.007	0.026±0.001	0.062±0.018	0.952±0.003	0.042±0.07
Inner Circuit HOMA	0.017±0	0.049±0.005	0.025±0.00	0.072±0.008	0.932±0.001	0.178±0.087
Mean Pyrrole HOMA	0.139±0.002	0.392±0.032	0.19±0.002	0.513±0.051	0.956±0.001	0.475±0.151
Total out of Plane	0.116±0.002	0.328±0.052	0.172±0.002	0.468±0.077	0.926±0.002	0.236±0.119

Feature Importance of LASSO model

Table S6: Feature importance for cone angles representation. Based on 10 bootstrap experiments with confidence intervals (α=0.05)

Property	Meso	Beta	Coordination	Axial	Metal
Absolute Ruffling	0.014±0.021	0.09±0.025	-0.142±0.016	-0.262±0.007	-0.261±0.008
Absolute Saddling	0.542±0.007	0.684±0.006	-0.254±0.011	0.063±0.009	-0.14±0.005
Total out of Plane	0.461±0.008	0.64±0.008	-0.302±0.011	-0.069±0.008	-0.226±0.006

Table S7: Feature importance for distance representation. Based on 10 bootstrap experiments with confidence intervals (α=0.05)

Property	Meso-Beta	Beta-Beta	Coordination	Axial	Metal
Absolute Ruffling	0.005±0.006	-0.072±0.007	-0.133±0.012	-0.263±0.007	-0.258±0.004
Absolute Saddling	-0.52±0.005	-0.297±0.006	-0.128±0.01	-0.052±0.009	-0.105±0.006
Total out of Plane	-0.401±0.006	-0.311±0.006	-0.189±0.011	-0.166±0.008	-0.199±0.006

Feature Importance of RF model

The random forest model was interpreted using impurity-based metric for feature importance as implemented in the Scikit-learn python package. The results are given in the following tables below. The importance scores of the random forest model agree very well with the ones of the LASSO model.

Table S8: Feature importance for cone angles representation. Based on 10 bootstrap experiments with confidence intervals (α=0.05)

Property	Meso	Beta	Coordination	Axial	Metal
Absolute Ruffling	0.249±0.008	0.245±0.005	0.114±0.009	0.206±0.006	0.186±0.006
Absolute Saddling	0.511±0.007	0.376±0.007	0.02±0.001	0.058±0.002	0.035±0.002
Total out of Plane	0.396±0.014	0.38±0.004	0.025±0.003	0.114±0.008	0.086±0.005

Table S9: Feature importance for distance representation. Based on 10 bootstrap experiments with confidence intervals (α=0.05)

Property	Meso-Beta	Beta-Beta	Coordination	Axial	Metal
Absolute Ruffling	0.251±0.007	0.243±0.007	0.117±0.008	0.201±0.007	0.187±0.006
Absolute Saddling	0.709±0.008	0.127±0.004	0.024±0.002	0.095±0.005	0.045±0.004
Total out of Plane	0.574±0.008	0.156±0.006	0.037±0.002	0.131±0.003	0.102±0.003

Full List of CCDC Structure IDs

Below the full list of CCDC structure IDs used in the database is given. A total of 425 structures were used.

LAXDIX	RIRNUB	PILWUC	POLPIR	KEFFOQ	JOCSEA
MUNJUA	HUWTEA	NACXIA	VAVRAN	FOKDOB	TIFCIX
AWIQEC	ADIQAI	BADREE	EREDUA	BORJEY	ZONXEF
DIVVIO	MAXVUG	ETISIM	OBOHAP	TORHEQ	ZUTQEK

TOBTUC	KEDGEG	DUQFAY	DIXXIS	LIRFOJ	FUGCIW
DECKII	HUWTAW	BEDCAO	MAXGIE	MORZIE	HAMDAA
YEFPEF	KACVOB	XEPZUO	IRINIH	HISFIY	SULDAE
VIMZUN	TEZRIC	PIZGEN	QAPWOX	AZIVEK	JUZSUS
JOVROB	WAFTED	DAFBOC	DOJPAV01	HETDAL	GONHAS
FEMQUJ	ICIBOM	GISJEX	ICIYEY	NAPJAR	RIGWUC
ATUSOX	FAMTEW	NUFNEH	PAGLUE	JOCRUP	XEDTAE
BAYDEL	WACREY	NEFTUN01	MAJMOD	CEHVIX	FEMQIX
HAZMUR	CEHVUJ	POLPEN	UTEYIE	INIBAL	MOKHIF
OBAFAB	LEZLAD	FOVTOC	SAHNAR	IWISAK	NANZEK
VOZDIZ	SOQYIG	MUQTUQ	CEZKEX01	IKACEE	IZABOA
MERPOP	DAHSUB	RUTQAY	GONHEW	ASIPUN	QAPXOY
ZEJLUX	EBORES01	VUMZAH	DAGSUC	VAFQEY	EBOMIS
QUNDUA	LIYFOO	NESHUO	TOCZAO	FOKDER	HALXIB
LOSGAB	NARDOA	QQQFQG01	YEQPOA	ZUZPIV	FEMQOD
MUHKAC	QAKJER	LIBCEE	VUMYEK01	XEYVUS	EREDIO
VEXSIC	NOHQEG	HOHZAG	UTEXID	KOXSAT	TEMFAS
IWOHEI	TEMKOL	GOBWAV	HUGXIT	KOGQUT	QUWTEJ
SOFYIY	QARCOD	BITPFE	MEWDAX	FIGVUM	HOHZEK
ECUBAH	NOKPEL	AWOMOP	DAFCIX	FAPCAC	LAXDAP
FAMTAS	LIYDEC	SOMDIJ	VAVRER	GIXCIB	OJIYEN
SULCUX	FAVGUE	IFASEQ	GISHUL	QAPWUD	
NIKPEC	ZESFUA	RUTMUO	GOWDAX	GOFNAT	
GECGEA	VOFDIE	TUBJAB	BIYXIS	NOVYEF	
HAMLAI	FOGGEP	IJURAI	ZEKXOE01	QAYRIS	
VAFQIC	PALFAL	LEFWOJ	QOYWAC	APANUC	
WEQBUQ	NUMTIA	HAMHIM	GOBBUV01	MEKTOL	
CAQNEO	LOKNAC	KIZSOC	RUTNEZ	HAMDII	
JUVXAB	AXUWUL	FAMTIA	SUNXUV	DAFCOD	
JOLKEC	TOJYID	GEQBOT	QIJXEM	KAXZOB	
KOXRUM	HOZKEN	QOQGUY	BUVKOV	WETKEN	
JAQROJ	HALWAS	PAZRAN	DUXROF	NOGWOX	
YOWJID	QOQHEJ	DOFXOL	XIXRII	XEZVED01	
EREFAI	EBOQUH	MOCSUS	QALJAS	XEFWIO	
QAPXEO	VAFTUR	VEFZOY	QIHYAJ	LUZYEK	
QAPXIS	ANUYOY	VEXTAV	USIVAV	JAWKOI	
NUMSOF	QAYRIS01	SOMDEF	YAJLUT	MOWPEW	
HUWSEZ	NIPNEG	SEBDOT	YESKOZ	ASIPOH	
CEBMAZ	HIZCEA	ROVCIQ	XEPTUI	QOSBAB	
HALWOG	MAXTUE	UGUHOV	NIFSUR	UFISUA	
BOHNUK	USULUR	QAYRIS02	WUTREJ	NTPOLC01	
VICDOB	CONQOL	ZOXQUA	ARODOC	RUTRAZ	
BUGJIX	IZAFIZ	NOKMIJ	DUVSOE	TAQWUG	

YEQPIU	XUZYUO	LIYFAA	HIZFAX	NIFTAY
EACWAG	NOLXUH	MUNKEL	VICDUH	EQUFON
NESJAW	EKETUJ	PODZEO	ODAWIZ	NOGWIR
IRINON	QOQJAH	VATXIX	TEVMOZ	VURJOK
SIMFAV	TEMFAS01	WIQJUE	ZEJMAE	IQOZET01
MOWPOG	LIYDUS	CIBTIS	TOCZES	EDOHIR
UMOQAP	ATEWUT	BUXVOG	KECJUA	OCIRID
ZEJMIM	YEHDAR	BORJOI	EWUSAS	EWURUL
XISYIH	NUKXEY	DEGQUD	JAVJIC	FOHYIL
YAJNAZ	BORKEZ	ALITEU	KUDLEB	KOJREG
UVUCOE	TPPMNA01	NAFNUG	XEPZOI	NUGMUA
QENJOI	BORJIC	MUGLIM	SEVCUR	XAPJAD
ETUKOW	TEVPIW	FATRAW	RUTPEB	WAXNOY
DITDES	EBORES	CAQMUF	BEGLUU	PUCPIM
CICQAG	ZEKXOE	QAKJUH	TEZREY	TEJXEP
GISJAT	XOFKIM	BUSFON	HOHZIO	ZEQRAO
RIHXOV	DEFKIM	XAMHIC	GOBBUV	LODSAY
OROJOV	LAXCOC	VOQPOG	NEMXOT	YEQMOW
AQOYAI	HAMGUX	QASPAC	LODSEC	ZITNIZ
CAHCOH	UVUCIY	VACQUL	RUTQIG	NEFTUN
CETWON	HISDUI	HEWDAO	FERWIK	AKOTUQ
HADHED	NOWXAB	WURQEF	NANZIO	CASNOC
DOWCAT	VICCUG	IWIRUD	SARROV	GISHIZ
MOWNUK	XEZVED	NEYWUM	ASIPIB	QAKJIV
LODRAX	DOKROM	EDOHAJ	TOFZET	FIRYOW
LAXBER	UQUQAA	BUFMUN	ROGSAK	REHGOB
NEYCAV01	ISAZEJ	ZITLUJ02	FOKDIV	NEYCAV
YASDOO	RIHXEL	UCIBUD01	JOCHOZ	VUMYUA
QEZGEI	ZEJMUY	UQIDAC	NEDCOO	NOKPAH
LOPCAW	SEMNED	MAJVEA	ZOHQOE	LAXCES
ZONQOI	UQIFEI	FARJIU	ZOHQUK	WOBGEZ
XUMPAY	IMELIV	REFPOH	LIYDOM	WOTQAZ
LARKEV	LODSIG	LEHSOI	ETISEI	TODXEP

Bibliography

[1] J. Krumsieck and M. Bröring, "*PorphyStruct*: A Digital Tool for the Quantitative Assignment of Non-Planar Distortion Modes in Four-Membered Porphyrinoids," *Chem. – Eur. J.*, vol. 27, no. 45, pp. 11580–11588, Aug. 2021, doi: 10.1002/chem.202101243.
